# Timing of Adjuvant Chemotherapy After Surgical Resection for Non-small Cell Lung Cancer: A Target Trial Emulation Using Nationwide Swedish Registry Data

**DOI:** 10.1245/s10434-025-17193-0

**Published:** 2025-03-19

**Authors:** Erik Lampa, Miklos Boros, Anders Berglund, Gunnar Wagenius, Gudrun N. Oskarsdottir

**Affiliations:** 1 Epistat AB, Uppsala, Sweden; 2 Linköping University Hospital, Linköping, Sweden; 3 Department of Oncology-Pathology Karolinska Institute, Stockholm, Sweden; 4 Thoracic Oncology Centre, Theme Cancer, Karolinska University Hospital, Stockholm, Sweden; 5 Department of Respiratory Medicine and Allergology, Skåne University Hospital, Lund, Sweden; 6 Division of Oncology, Department of Clinical Sciences Lund, Lund University, Lund, Sweden

**Keywords:** Lung cancer, Target trial emulation, Immortal-time bias, Real-world data

## Abstract

**Background:**

We investigated the impact of time to adjuvant chemotherapy (AC) on survival after surgical resection (<8 weeks or 8–16 weeks) for patients with non-small cell lung cancer (NSCLC) by applying a target-trial emulation.

**Material and Methods:**

We used Swedish population-based healthcare registries to emulate a hypothetical target trial, with treatment arms of ‘initiate AC <8 weeks postoperatively’ and ‘initiate AC 8–16 weeks postoperatively’. The clone-censor-weight approach was used in which all patients were cloned and all clones were assigned to each treatment arm. Clones were then censored when the assigned treatment was no longer compatible with the actual treatment.

**Results:**

We included 510 patients in the hypothetical target trial, of whom 51% received AC and 150 (57%) started AC within 8 weeks. More than half of the patients were female (52.5%) and the mean age was 69 years. The 5-year disease-free survival (DFS) in the emulated trial for the group who initiated AC <8 weeks postoperatively was 50.3% and the 5-year overall survival (OS) was 58.1%. For the group who initiated AC 8–16 weeks postoperatively, the emulated trial showed a 5-year DFS and OS of 49.1% and 57.1%, respectively.

**Conclusion:**

By using target trial emulation, our study supports earlier data on timing for AC after surgical resection for NSCLC. However, further research is needed and our data indicate that a randomized controlled trial could be conducted without major harm to the experimental group (>8 weeks).

Lung cancer is the second most common cancer diagnosed in the world, and approximately 4500 patients are diagnosed each year in Sweden.^1^ For patients with early-stage disease that is operable, surgical resection is the optimal treatment.^2^ In Sweden, approximately 27% of all lung cancer patients undergo surgical resection as the first treatment.^1^

In 2008, a study by the LACE (Lung Adjuvant Cisplatin Evaluation) Collaborative Group was published that proved the increased survival of patients with non-small cell lung cancer (NSCLC) who received adjuvant cisplatin-based chemotherapy (AC).^3^ This study, along with other similar studies,^4,5^ showed that patients with higher TNM stage had better effect, leading to a recommendation of considering adjuvant chemotherapy for patients with stages IB and higher in many guidelines.^2,6^

Newer studies have shown an important role for immunotherapy in the neoadjuvant or adjuvant setting,^7,8^ as well as targeted therapy for patients with targetable mutations;^9^ therefore, this is likely to be the new standard treatment for many patients. However, there will always be some patients who have contraindications for immunotherapy and therefore it is important to continue to research the best way to administer chemotherapy as an adjuvant/neoadjuvant treatment.

Most guidelines recommend starting AC within 8 weeks postoperatively,^2,6^ and retrospective analyses have supported this.^10^ A randomized controlled trial (RCT) that randomized patients to either start treatment within 8 weeks or start treatment later than 8 weeks is trivial to answering the question of treatment initiation timing; however, in the absence of RCTs, observational data may be used to emulate the ideal RCT—the target trial.^11^ Emulating a target trial does not transform the observational data into an RCT as the observational data still contain sources of bias, most notably non-random treatment assignment. One key issue when emulating a target trial is the misalignment of eligibility, start of follow-up, and treatment assignment.^11^ In an RCT, treatment assignment is made at randomization but patients receiving AC rarely do so at the time of surgery. In observational data, initiation of AC is thus only observed for patients who were alive at the date of treatment initiation. Ignoring this fact leads to immortal time bias, which biases the treatment effect upwards. A proposed strategy to mitigate this bias consists of creating exact copies of each patient in the data (cloning) and assign each clone to one of the strategies. As soon as a clone’s data are no longer consistent with the assigned treatment, that clone is censored.^11^ For example, if a clone is assigned to initiate AC within 8 weeks but starts therapy at 9 weeks, then the clone is censored at week 8. This creates artificial censoring and needs to be adjusted for; for example, by weighting. Application of the clone-censor-weight methodology in oncology is not new. Maringe et al. provided a tutorial on the methodology and investigated the effects of surgery on timing in elderly lung cancer patients.^12^ Furthermore, Petito et al. emulated two target trials using the Surveillance, Epidemiology, and End Results-Medicare linked database to estimate the effectiveness of adding a drug to the treatment regimen in elderly patients with colorectal cancer.^13^

To be able to have as reliable data as possible, complete register data are needed. The Swedish National Lung Cancer Registry (NLCR) has almost complete coverage of the Swedish lung cancer population, with over 97% of patients being registered. The individual patient overview (IPÖ) in the NLCR contains detailed follow-up data on all patients regarding treatments, healthcare visits, and response assessment.

Overall, we know that adjuvant chemotherapy will continue to be a mainstay in treatment for early-stage lung cancer, even if combinations with other treatments are most likely becoming standard of care. Using high-quality data, we emulated a target trial on a nationwide population of lung cancer patients, and were able to obtain better guidance on what timing is best for adjuvant postoperative chemotherapy in terms of overall survival (OS) and disease-free survival (DFS).

## Material and Methods

### Study Design and Participants

This was a retrospective, nationwide cohort study using existing data collected from the NLCR in Sweden. Patients over 18 years of age with incident stages IB–IIIA who underwent surgical resection between 1 January 2014 and 31 August 2017 were included to ensure adequate follow-up time. Patients were stratified into two groups: those receiving AC <8 weeks postoperatively or those receiving AC 8–16 weeks postoperatively. The index date was set at the time of operation and stopped at progression, death, or 5 years postoperatively.

To be able to emulate the trial, we first had to identify how we would design the theoretical trial—the target trial. In Table 1 we describe the target trial and how we emulated the trial using data from the NLCR.

**Table 1:** A possible protocol for a hypothetical target trial to answer whether the timing of adjuvant chemotherapy affects survival of early-stage NSCLC patients

Protocol component	Target trial	Emulated trial using lung cancer registry
Eligibility criteria	Patients with stage IB–IIIA NSCLC who have had surgical resection without neoadjuvant treatment, PS 0–2	Patients aged 18 years and over with stage IB–IIIA NSCLC who have had surgical resection without neoadjuvant treatment, between 1 January 2014 and 31 August 2017, PS 0–2
Treatment strategies	Adjuvant chemotherapy before 8 weeks or 8–16 weeks postoperatively	Adjuvant chemotherapy is defined as initiating chemotherapy postoperatively containing either cisplatin, carboplatin, or vinorelbine. The treatment strategies are defined as: • initiate adjuvant chemotherapy before 8 weeks postoperatively • initiate adjuvant chemotherapy between 8 and 16 weeks postoperatively
Assignment procedures	Participants will be randomly assigned to either strategy at baseline and will be aware of the strategy to which they have been assigned	Treatment is not randomly assigned
Follow-up period	Starts at randomization and ends at diagnosis of recurrent disease, death, loss to follow-up, or 5 years after baseline, whichever occurs first	Starts at date of surgical resection and ends at diagnosis of recurrent disease, death, loss to follow-up, or 5 years after baseline, whichever occurs first
Outcome	Disease-free survival: Lung cancer recurrent disease diagnosed by a healthcare professional, or death due to any cause within 5 years of baseline	Disease-free survival: Lung cancer recurrent disease diagnosed by a healthcare professional, or death due to any cause within 5 years of baseline
	Overall survival: Death due to any cause within 5 years of baseline	Overall survival: Death due to any cause within 5 years of baseline
Causal contrasts of interest	Absolute difference in 5-year disease-free and overall survival between the treatment groups. RMST difference at 5 years	Absolute difference in 5-year disease-free and overall survival between the treatment groups. RMST difference at 5 years
Analysis plan	Disease-free and overall survival estimated using the Kaplan–Meier estimator	Analyses conducted on a cloned dataset assuming random treatment assignment with a censoring indicator when the treatment assignment deviates from the protocol. IPCW is used to account for selection bias. Cox regression models for the IPCW will be adjusted for tumor stage, performance status, and healthcare region. Five-year disease-free and overall survival is estimated using an IPCW-weighted pooled logistic regression model, adjusted for age, sex, tumor stage, baseline performance status, healthcare region, and smoking status. The average treatment effect will be estimated using a G-computation estimator. The nonparametric bootstrap is used to estimate a 95% confidence interval for the survival and RMST differences

To emulate such a trial, the material on the right column was used for this study

NSCLC, non-small cell lung cancer; PS, World Health Organization performance status; IPCW, inverse probability of censoring weights; RMST restricted mean survival time

### Treatment Strategies

#### Statistical Methods

As we used a trial emulation approach, a dataset with two copies (clones) of every eligible individual was created and each clone was assigned to one of the treatment strategies at baseline. Adherence to the treatment strategies was checked each week and clones were censored if and when the assigned treatment strategy deviated from the actual treatment. Clones assigned to the ‘initiate adjuvant chemotherapy <8 weeks postoperatively’ strategy were censored at the end of the seventh week if they had not initiated adjuvant chemotherapy by that week. Clones assigned to the ‘initiate AC 8–16 weeks postoperatively’ strategy were censored at the week they initiated treatment if they initiated treatment prior to 8 weeks, or at 16 weeks if they had not initiated treatment at the end of the 15th week. Although we could not estimate the observational analog to the intention-to-treat effect, the treatment strategies required individuals to initiate treatment within 8 or 16 weeks regardless of future adherence; this approach is similar to the intention-to-treat effect.

To adjust for the selection bias caused by artificial censoring, each clone was assigned a time-varying inverse probability of censoring weight (IPCW). Stabilized IPCWs were estimated using methods described elsewhere.^14,15^ The weights are the cumulative product of predictions from two Cox regression models. The numerator model contained TNM stage at surgery, performance status at surgery, healthcare region, and the patient’s age, modeled using restricted cubic splines with knots at the 5th, 35th, 65th, and 95th percentiles. The denominator model included all variables in the numerator model plus the performance status from the previous week as a time-varying variable. The rationale for including the time-varying performance status is that patients may be too frail to start AC within 8 weeks. While we lack data on, for example, comorbidity scores, performance status serves as a proxy for frailty.

The outcome model was an IPCW logistic regression model, incorporating treatment arm, time modeled using restricted cubic splines with knots at the 5th, 35th, 65th, and 95th percentiles, and their interaction, as well as the variables from the numerator model. The resulting estimate of the treatment effect is a conditional effect rather than a marginal effect. Marginal survival curves were estimated by standardizing the associations according to the covariate distribution in the data.^16^

The nonparametric bootstrap was used to obtain 95% confidence intervals (CIs) by sampling patients with replacement and performing all analysis steps in each of the 2000 bootstrap resamples. All analyses were performed using R version 4.3.2 (The R Foundation for Statistical Computing, Vienna, Austria), using the survival, Epi, boot and ipw add-on packages.

#### Ethics

This study was conducted in accordance with the International Society for Pharmacoepidemiology (ISPE) Guidelines for Good Pharmacoepidemiology Practices (GPP) and was approved by the Swedish Ethical Review Authority (Etikprövningsmyndigheten). Informed patient consent was not required due to the retrospective nature of the study.

## Results

### Patient Population and Treatment Patterns

A total of 510 patients fulfilled the inclusion criteria. Patient demographics are shown in Table 2. The median age of patients was 69 years (interquartile range [IQR] 64–74) and more than half of the patients were female (52.5%).

**Table 2:** Demographics of the patients^a^ operated for NSCLC in the years 2014–2017 and registered in the Swedish National Lung Cancer Registry stratified by actual treatment received

	Overall	No AC	AC <8 weeks	AC 8–16 weeks
*N*	507	246	149	112
Age, years (median [IQR])	69.00 [64.00–74.00]	70.00 [66.00–75.00]	67.00 [61.00–71.00]	69.00 [63.00–74.00]
Women	270 (53.3)	134 (54.5)	84 (56.4)	52 (46.4)
*TNM stage*				
IB	214 (42.2)	118 (48.0)	59 (39.6)	37 (33.0)
IIA	125 (24.7)	53 (21.5)	37 (24.8)	35 (31.2)
IIB	103 (20.3)	48 (19.5)	28 (18.8)	27 (24.1)
IIIA	65 (12.8)	27 (11.0)	25 (16.8)	13 (11.6)
*WHO PS*				
0	265 (52.3)	115 (46.7)	90 (60.4)	60 (53.6)
1	217 (42.8)	112 (45.5)	54 (36.2)	51 (45.5)
2	25 (4.9)	19 (7.7)	5 (3.4)	1 (0.9)
*Smoking status*				
Smoker	200 (39.4)	96 (39.0)	56 (37.6)	48 (42.9)
Former smoker	250 (49.3)	118 (48.0)	76 (51.0)	56 (50.0)
Never smoker	57 (11.2)	32 (13.0)	17 (11.4)	8 (7.1)
*Healthcare region*				
Stockholm/Gotland	81 (16.0)	37 (15.0)	24 (16.1)	20 (17.9)
Uppsala/Örebro	85 (16.8)	44 (17.9)	26 (17.4)	15 (13.4)
South-East	118 (23.3)	82 (33.3)	16 (10.7)	20 (17.9)
South	36 (7.1)	15 (6.1)	12 (8.1)	9 (8.0)
West	81 (16.0)	26 (10.6)	25 (16.8)	30 (26.8)
North	106 (20.9)	42 (17.1)	46 (30.9)	18 (16.1)
*Type of surgery*				
Wedge resection	18 (3.6)	10 (4.1)	6 (4.1)	2 (1.8)
Segmentectomy	8 (1.6)	8 (3.3)	0 (0.0)	0 (0.0)
Lobectomy	430 (86.0)	210 (86.4)	122 (84.1)	98 (87.5)
Pneumonectomy	41 (8.2)	15 (6.2)	15 (10.3)	11 (9.8)
Bilobectomy	2 (0.4)	0 (0.0)	1 (0.7)	1 (0.9)
Unknown	1 (0.2)	0 (0.0)	1 (0.7)	0 (0.0)
*Histology*				
Adenocarcinoma	344 (67.9)	160 (65.0)	106 (71.1)	78 (69.6)
Large cell carcinoma	18 (3.6)	14 (5.7)	3 (2.0)	1 (0.9)
Squamous cell NSCLC	131 (25.8)	67 (27.2)	34 (22.8)	30 (26.8)
NSCLC, not otherwise specified	14 (2.8)	5 (2.0)	6 (4.0)	3 (2.7)

Data are expressed as *n* (%) unless otherwise specified

AC, adjuvant chemotherapy; IQR, interquartile range; TNM, tumor, node, metastasis; WHO PS, World Health Organization performance status; NSCLC, non-small cell lung cancer

^a^Race/ethnicity data are not available in the register. Statistic Sweden report 80% of the population being born in Sweden

**Fig. 1:**
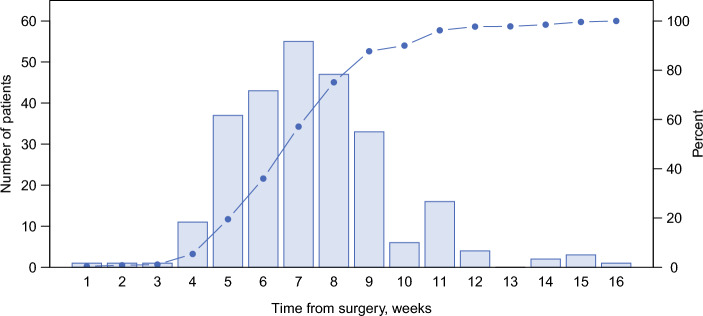
AC initiation by week postoperatively. The bars show the total number of patients having received AC at each timepoint, and the whole line shows the ratio of patients who have received treatment at each dot. Week *n* means that the patient initiated AC between weeks *n* –1 and week *n*. *AC* adjuvant chemotherapy

Most patients were stage IB (42.2%) and more than half received AC (51.6%). Approximately 90% of patients were smokers or former smokers and 11.2% had never smoked. There was a regional difference in the number of patients from different regions in Sweden; most patients were from the South East healthcare region (23.3%), while the South region had had fewest patients (7.1%). For patients receiving adjuvant chemotherapy, 57% (150 patients, 29% of the total patients) received treatment within 8 weeks of the surgical date, whereas 113 patients received the treatment 8–16 weeks postoperatively. The TNM stage differed between patients receiving AC within 8 weeks and those receiving AC between 8 and 16 weeks, with the former group consisting of a higher proportion of patients with stage IB and IIIA (39.6% vs. 33.0%, and 16.8 vs. 11.6%, respectively). Consequently, the proportion of patients with stage IIA or IIB was lower among patients receiving AC within 8 weeks relative to those receiving AC within 8–16 weeks (24.8% vs. 31.2% and 18.8% vs. 24.2%, respectively). Patients receiving AC within 8 weeks consisted of fewer current smokers (37.6%) than patients receiving AC between 8 and 16 weeks (42.9%). Distribution of AC timing for patients after surgery is shown in Fig. 1. The majority of patients initiated AC within 8 weeks, and by 10 weeks, 241 patients (90.3%) had initiated treatment. Table 3 shows the surgical modalities for each stage stratified by the actual treatment received. The majority of the surgeries were lobectomies, but the proportion of pneumonectomies increased at higher stages (between 1.7% and 5.4% in stage IB to between 14.8% and 16% in stage IIIA), although the actual numbers were small.

**Table 3:** Surgical modalities by stage of disease, stratified by actual treatment received

Stage	Type of surgery	No AC	AC <8 weeks	AC 8–16 weeks
IB	Wedge resection	7 (6.0)	2 (3.6)	0 (0.0)
	Segmentectomy	5 (4.3)	0 (0.0)	0 (0.0)
	Lobectomy	103 (88.0)	51 (91.1)	36 (97.3)
	Pneumonectomy	2 (1.7)	3 (5.4)	1 (2.7)
	Bilobectomy	0 (0.0)	0 (0.0)	0 (0.0)
	Unknown	0 (0.0)	0 (0.0)	0 (0.0)
IIA	Wedge resection	0 (0.0)	0 (0.0)	1 (2.9)
	Segmentectomy	1 (2.0)	0 (0.0)	0 (0.0)
	Lobectomy	47 (92.2)	30 (83.3)	30 (85.7)
	Pneumonectomy	3 (5.9)	4 (11.1)	4 (11.4)
	Bilobectomy	0 (0.0)	1 (2.8)	0 (0.0)
	Unknown	0 (0.0)	1 (2.8)	0 (0.0)
IIB	Wedge resection	2 (4.2)	1 (3.6)	0 (0.0)
	Segmentectomy	1 (2.1)	0 (0.0)	0 (0.0)
	Lobectomy	39 (81.2)	23 (82.1)	22 (81.5)
	Pneumonectomy	6 (12.5)	4 (14.3)	4 (14.8)
	Bilobectomy	0 (0.0)	0 (0.0)	1 (3.7)
	Unknown	0 (0.0)	0 (0.0)	0 (0.0)
IIIA	Wedge resection	1 (3.7)	3 (12.0)	1 (7.7)
	Segmentectomy	1 (3.7)	0 (0.0)	0 (0.0)
	Lobectomy	21 (77.8)	18 (72.0)	10 (76.9)
	Pneumonectomy	4 (14.8)	4 (16.0)	2 (15.4)
	Bilobectomy	0 (0.0)	0 (0.0)	0 (0.0)
	Unknown	0 (0.0)	0 (0.0)	0 (0.0)

AC, adjuvant chemotherapy

Table 4 shows the estimated 5-year DFS, restricted mean survival time (RMST), and hazard ratios (HRs) for all patients. In the naïve analysis, the 5-year DFS for the group who started AC <8 weeks postoperatively was 50.0% (95% CI 42.6–58.7), and 46.8% (95% CI 38.4–57.1) for the group who started AC 8–16 weeks postoperatively, giving an estimate of the benefit of AC within 8 weeks of 3.2% (95% CI −15.4 to 9.1). The difference in RMST was estimated to be 3.2 months (95% CI −8.5 to 2.0), indicating a benefit of AC within 8 weeks postoperatively. The 5-year DFS for the group that did not initiate AC was 46.3% (95% CI 40.5–53.0). In the emulated trial, the 5-year DFS was estimated as 50.3% (95% CI 42.9–61.7) for the group who started AC <8 weeks postoperatively and 49.1% (95% CI 40.2–62.5) for the group who started AC 8–16 weeks postoperatively. The difference between the groups was lower compared with the naïve analysis, i.e. 1.1% (95% CI −14.1 to 11.3) in favor of AC within 8 weeks. The RMST was also attenuated towards zero, i.e. 1.8 months (95% CI −7.2 to 3.4) in favor of AC within 8 weeks. The same information for OS is shown in Table 5, with the OS estimates (5-year risk difference and RMST) being similarly attenuated towards zero compared with the naïve analysis, i.e. 1.0% (95% CI −13.9 to 10.7) and 1.0 months (95% CI −5.6 to 3.3) vs. 4.4% (95% CI −16.6 to 7.8) and 2.0 (95% CI −6.7 to 2.6) in favor of AC within 8 weeks. The 5-year OS for the group that did not initiate AC was 52.4% (95% CI 46.6–59.1). Using the group who started AC within 8 weeks as the reference group, HRs were 1.1 (95% CI 0.8–1.6) and 1.2 (95% CI 0.8–1.7) in the naïve analyses for DFS and OS, respectively. These estimates were attenuated to 1.0 (95% CI 0.8–1.3) and 1.0 (95% CI 0.8–1.4), respectively, in the emulated trial. Although all differences favored AC within 8 weeks, the results were not significant at conventional thresholds.

**Table 4:** Estimated 5-year DFS, RMST, and HR according to the naïve analysis and the emulated trial

	5-year DFS (95% CI), %	RMST (95% CI), months	HR (95% CI)
*Naïve analysis*			
Adjuvant chemotherapy <8 weeks	50.0 (42.6–58.7)	42.8 (39.5–46.2)	1 (reference)
Adjuvant chemotherapy 8–16 weeks	46.8 (38.4–57.1)	39.6 (35.6–43.6)	1.1 (0.8–1.6)
Difference	−3.2 (−15.4 to 9.1)	−3.2 (−8.5 to 2.0)	
*Emulated trial*			
Adjuvant chemotherapy <8 weeks	50.3 (42.9–61.7)	44.7 (41.3–49.7)	1 (reference)
Adjuvant chemotherapy 8–16 weeks	49.1 (40.2–62.5)	42.8 (38.8–49.0)	1.0 (0.8–1.3)
Difference	−1.1 (−14.1 to 11.3)	−1.8 (−7.2 to 3.4)	

DFS, disease-free survival; RMST, restricted mean survival time; HR, hazard ratio; CI, confidence interval

**Table 5:** Estimated 5-year OS, RMST, and HR according to the naïve analysis and the emulated trial

	5-year OS (95% CI), %	RMST (95% CI), months	HR (95% CI)
*Naïve analysis*			
Adjuvant chemotherapy <8 weeks	57.0 (49.6–65.6)	47.4 (44.5–50.4)	1 (reference)
Adjuvant chemotherapy 8–16 weeks	52.7 (44.2–62.8)	45.4 (41.8–49.0)	1.2 (0.8–1.7)
Difference	−4.4 (−16.6 to 7.8)	−2.0 (−6.7 to 2.6)	
*Emulated trial*			
Adjuvant chemotherapy <8 weeks	58.1 (51.3–69.2)	50.5 (48.2–54.5)	1 (reference)
Adjuvant chemotherapy 8–16 weeks	57.1 (48.1–69.2)	49.5 (46.2–54.1)	1.0 (0.8–1.4)
Difference	−1.0 (−13.9 to 10.7)	−1.0 (−5.6 to 3.3)	

OS, overall survival; RMST, restricted mean survival time; HR, hazard ratio; CI, confidence interval

**Fig. 2:**
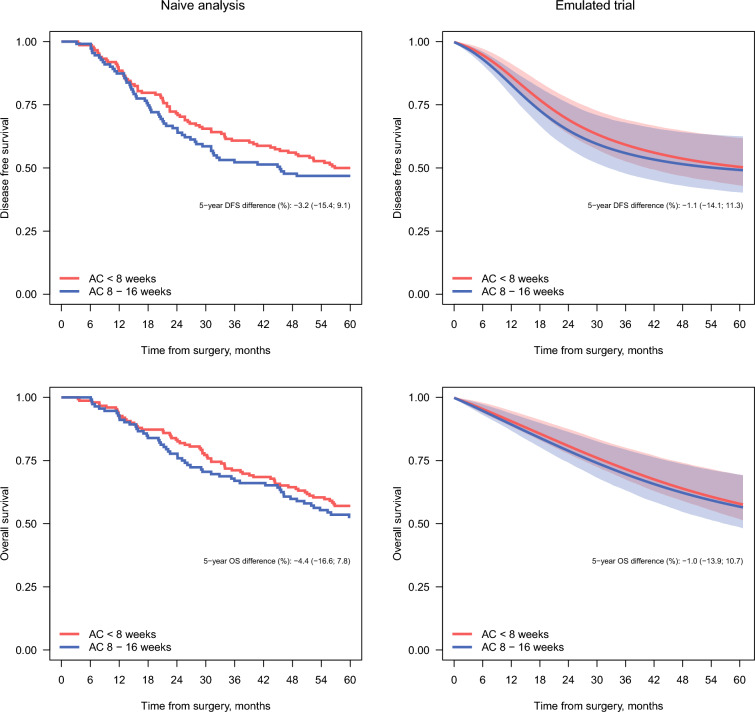
DFS (top row) and OS (bottom row) for non-small cell lung cancer patients receiving adjuvant chemotherapy before versus after 8 weeks postoperatively, with (naïve analysis) and without (emulated trial) emulating a trial. *DFS* disease-free survival, *OS* overall survival, *chemo* chemotherapy

Figure 2 displays Kaplan–Meier curves for DFS (top left panel) and OS (bottom left panel), as well as marginal survival curves for DFS (top right panel) and OS (bottom right panel). Visually, it looks like survival is worse for the group who started AC 8–16 weeks postoperatively, and more so for DFS than OS, although the data do not support any differences between the curves.

## Discussion

Using data from the NLCR in Sweden, we emulated a trial to provide further insight into the optimal timing of AC for patients with NSCLC. The current guidelines recommend introducing AC within 8 weeks postoperatively.^10,17^ Our naïve data show a trend towards better survival for patients in the group that received AC within 8 weeks postoperatively. After emulating a target trial, the difference between the group that received AC within <8 weeks and the group that received AC 8–16 weeks postoperatively decreased even though there was still a trend towards better survival for the group that received AC within <8 weeks postoperatively. We therefore believe that our data support the earlier evidence of time to AC, recommended within 8 weeks. Nevertheless, this study relies on retrospective data and an RCT is needed to prove this.

The current study is important due to the fact that no prospective studies have been conducted to assess this topic; however, further studies are needed to support the current guidelines. There is a good theoretical rationale as to why AC should be started as early as possible after curative surgical resection. Other studies have suggested that there is an increase in circulating tumor cells postoperatively that could potentiate the growth of metastasis.^18^ In other tumor types such as breast cancer and colorectal cancer, later administration of AC has been shown to have a negative effect on survival.^19,20^ This effect has not been proved in NSCLC and the reason for this is unclear. The consensus in guidelines is to strive towards starting treatment with AC as early as possible.^2^ Clinical studies have shown the benefit of AC, hence that is not debatable^3^ even if the effect is marginal. By using the method of emulating a target trial and using the clone-censor-weight approach, the results can hypothetically be similar to a prospective trial.^13^

Salazar et al. previously published a retrospective study involving a large cohort of patients (*n* = 12,473) to address the same question.^10^ These researchers used Cox models, restricted cubic splines, and the National Cancer Database, a hospital-based tumor registry that includes over 70% of lung cancer cases in the United States. We used a different methodology that we believe adds to the knowledge previously shown by others, such as Salazar et al. Our results agree with the conclusion that AC can be used at different time points but is likely most effective if applied as soon as possible. In our current study, we used the Swedish NLCR, a database with over 97% coverage of all lung cancer cases in the country. Therefore, the data in our study should cover all patients with NSCLC that are operated in Sweden during these years, with the exception of only a very few cases.

In addition, we saw a certain regional difference in the administration of AC in Sweden. All centers in Sweden follow the same national guidelines on lung cancer treatment,^6^ but as in other diseases, the interpretation of these guidelines can differ and can therefore be the cause of the different numbers we see in this study. We believe this is interesting and are planning more studies within the national lung cancer registry (including socioeconomical factors, etc.) to further explain these differences.

AC has already been shown to be a treatment that increases the survival of patients with NSCLC stages II and higher, and, in some cases, stage IB.^3^ This has been known for many years and discussing this is not the purpose of our study, as it is not designed to do so. The power of our study would not be sufficient and our goals are different.

Newer therapies, such as immunotherapy and targeted therapy, in combination with chemotherapy, have now shown better overall results than chemotherapy alone.^7,9,21–23^ These treatments have therefore become the mainstay of therapy for these patients. Some patients have contraindications to immunotherapy and therefore AC will continue to be a treatment used for this group. We believe it is important to continue to improve the evidence on this treatment as long as it is in use, and, at the same time, we are finding improved methods for increasing survival.

Most patients in our cohort received AC within the recommended treatment window of 8 weeks. Those who received AC later than 8 weeks mostly had treatment 1 or 2 weeks after that, with the frequency of treatment decreasing as time passed, as expected. It can be argued that we should have had smaller interval groups. However, we believe that the important timeline was at 8 weeks, as the guideline stated this timeline as a reference point; therefore, we decided on two groups. In our ‘real world’ data, it is clear that the guidelines are quite thoroughly followed; however, as expected, some patients fall outside the recommended guidelines. Therefore, it is possible to apply the statistical method we used to emulate a target trial. We started the inclusion process when the follow-up database at the register (IPÖ) was created, and in order to have a 5-year follow-up, the last year of inclusion was 2017.

Investigation of the timing of adjuvant chemotherapy is a classic example of where the results can be influenced by immortal time bias. A recent study by Kirkegård et al. investigated the timing of adjuvant chemotherapy treatment after pancreatic cancer surgery (0–4 weeks or 4–8 weeks after discharge). They concluded that survival was not significantly different between early versus late initiators.^24^ Patients receiving treatment later than 8 weeks postoperatively must be alive to receive treatment and one would expect a survival benefit the longer a patient had to wait for their treatment. This was not the case in the naïve analysis, as initiating treatment <8 weeks postoperatively showed a small benefit, suggesting that the results were not heavily influenced by immortal time bias but possibly that the group receiving treatment >8 weeks postoperatively consisted of more frail patients. The IPCW tried to adjust for this and the small DFS benefit was halved in the trial emulation.

The strengths of our study were that we included a nationwide cohort with good coverage, as well as showing real-world data. There were also several limitations to our study, including its small sample size and the retrospective nature of the data. The CIs are wide and our study cannot rule out the harmful effects of AC treatment <8 weeks postoperatively compared with >8 weeks postoperatively. Hernán^25^ stated that it is preferable to have multiple studies with imprecise estimates than having no study at all. In the absence of an RCT, we encourage others to investigate this question and provide estimates that can be meta-analyzed to arrive at a more precise estimate of the risk differences of AC timing on OS and DFS.

Due to the retrospective nature of this study, we included an important confounding of indication—the reasons for starting chemotherapy at a given time. This can only be eliminated by conducting an RCT, which is needed to confirm our data as our study alone cannot be used as a clinical guideline. Since 2017 (the date of our last inclusion) much has changed in lung cancer treatment, such as segmentectomies being more commonly used as a surgical method and the increasing use of video-assisted thoracoscopies (VATS) instead of thoracotomies.

## Conclusion

Our key findings in this retrospective study can be summarized as follows: later administration of AC did not decrease the DFS or OS of patients, and by emulating a target trial using the clone-censor-weight approach, the difference in the survival of patients decreased, excluding immortal time bias. The question of optimal timing of AC needs to be studied further. Our conclusions support the fact that an RCT can be conducted safely, without major harm to the experimental group (>8 weeks).

## Data Availability

The data from this study can be obtained by contacting and discussing with the steering board of the NLCR.
